# Insights into frontline healthcare workers’ mental health amidst COVID-19 – Sources of workplace worries and coping strategies

**DOI:** 10.1016/j.heliyon.2023.e20258

**Published:** 2023-09-18

**Authors:** Halim Ismail, Yong-Hong Phan, Kausalyaa Chandriah, Mohd Ridzwan Arman, Nurul Nabihah Mokhtar, Siti Aishah Hamdan, Sheng Qian Yew

**Affiliations:** Department of Public Health Medicine, Faculty of Medicine, Universiti Kebangsaan Malaysia, Jalan Yaacob Latif, Bandar Tun Razak, 56000, Cheras, Kuala Lumpur, Malaysia

**Keywords:** Occupational mental disorders, Workplace worries, Coping strategies, Health care workers, COVID-19

## Abstract

**Introduction:**

This study was aimed to measure the prevalence of depression, anxiety, and stress as well as to evaluate the associations of these mental disorders with sociodemographic factors, workplace worries, and coping strategies among frontline HCWs in Kuala Lumpur, Malaysia during the COVID-19 pandemic.

**Methods:**

A cross-sectional study was conducted in a tertiary teaching hospital in Kuala Lumpur, Malaysia. Sociodemographic data questionnaire, Depression, Anxiety, and Stress Scale 21, sources of workplace worries questionnaire, and Brief-COPE inventory were randomly distributed to frontline HCWs who worked at the medical, emergency, and anaesthesiology departments. Data were analyzed using Chi-square tests and multivariable linear regression analysis.

**Results:**

A total of 137 frontline HCWs responded to the questionnaires. The prevalence of depression, anxiety, and stress was 69.3%, 77.4%, and 57.7%, respectively. None of the sociodemographic characteristics was associated with depression, anxiety, and stress. Depression was associated to all sources of workplace worries, except “fear of getting infected” (p = 0.089), while anxiety and stress were associated with all sources of workplace worries. Humour (β = 0.821), self-blame (β = 0.686), denial (β = 0.676), substance use (β = 0.835), and behavioural disengagement (β = 0.583) were positively correlated to depression. However, active coping (β = −0.648) and acceptance (β = −0.602) were negatively correlated to depression. On the other hand, active coping (β = 0.913), planning (β = 0.879), acceptance (β = 0.831), religion (β = 0.704), and self-distraction (β = 0.929) were positively correlated to stress. Only substance use (β = −0.417) was negatively correlated to stress. All coping strategies did not correlate to anxiety.

**Conclusion:**

The high prevalence of depression, anxiety and stress is attributed by the various sources of workplace worries and the inappropriate coping strategies among the frontline HCWs. Measures that minimise workplace worries and inappropriate coping strategies must be implemented promptly.

## 1 Introduction

COVID-19 is the largest communicable disease outbreak that Malaysia has ever experienced since the outbreaks of Spanish Flu [1], Nipah virus [2], and SARS-CoV [3], in 1918, 1999, and 2003, respectively. According to the World Health Organization (WHO), Malaysia has reported a total of 5,108,586 confirmed cases of COVID-19 till July 2023, with 37,110 mortalities since the outbreak of the disease. The period prevalence of COVID-19 infection and the mortality rate among health care workers (HCWs) in Malaysia were 1.03% and 0.0019%, respectively [4]. Although these figures appear to be relatively low, it is alarming that a majority of COVID-19 infections among the HCWs in Malaysia was originated from the workplace (53.3%), in which 36.3% of cases were due to cross-infection between HCWs and 17.0% of cases were contracted from the patients under their care. Not surprisingly, HCWs had a 2.9 times higher incidence risk ratio for the acquisition of COVID-19 infection than the general population [4].

On top of the increased risk of COVID-19 infection, HCWs are also more likely to suffer from depression, anxiety, and stress during the pandemic. In their review, Salari et al. reported that the prevalence of depression, anxiety, and stress among HCWs was 24.3%, 25.8%, and 45%, respectively [5], which was significantly higher than the pre-pandemic era. This statement was later agreed by the WHO, who announced a 27.6% increase in depression and 25.6% increase in the cases of anxiety among HCWs worldwide during the pandemic [6]. This observation can be explained by the increased exposure of HCWs to various occupational hazards during the pandemic. For instance, HCWs are more severely affected by biological hazard (i.e., SARS-CoV-2) [7], psychological hazard (due to increased workload, stressors, working hours, and staff shortages) [8], physical hazard (due to prolonged use of personal protective equipment) [9], and chemical hazard (due to of prolong exposure to disinfectant) while delivering quality health care at their workplace [10]. All these occupational hazards could collectively increase the risk of mental health disorders among the HCWs, which in turn exert detrimental impacts on the healthcare system. A recent review reported that HCWs who experience mental health disorders may exhibit reduced levels of professionalism, leading to the delivery of lower-quality care, decreased efficiency, and an elevated risk of committing medical errors. These outcomes collectively pose a serious threat to patients’ well-being and safety [11].

Although the prevalence and the determinants of depression, anxiety, and stress among HCWs has been extensively investigated during the COVID-19 pandemic [[12], [13], [14], [15]], these studies were mostly conducted among general HCWs (i.e., they included all clinical and non-clinical hospital staff across all departments) but not among the frontline HCWs. Frontline HCWs are defined as healthcare providers who are specifically engaged in the direct diagnosis, treatment, and care of COVID-19 positive patients during the pandemic. As a result, these findings may not portray the real situation faced by the frontline HCWs specifically, who constantly suffer from a higher risk of COVID-19 infection, heavier workloads, longer shifts, and social stigma. In addition, when examining the level of depression, anxiety, and stress, most of these studies did not take the workplace worries and coping strategies into account, given that these are the established factors that will affect depression, anxiety, and stress.

To address these limitations, the present study was aimed: (i) To measure the prevalence of depression, anxiety, and stress among frontline HCWs in Kuala Lumpur, Malaysia during the COVID-19 pandemic; (ii) To determine the association between sociodemographic characteristics and depression, anxiety and stress; (iii) To determine the association between the sources of workplace worries and depression, anxiety and stress; and (iv) To identify the correlation between the various coping strategies with depression, anxiety, and stress among the frontline HCWs.

## 2 Materials and methods

### 2.1 Study design and study setting

A cross-sectional design was applied in the present study. The study was conducted in a teaching hospital in Kuala Lumpur, Malaysia, which is a major referral hospital that provides tertiary care for COVID-19 positive patients throughout the pandemic.

### 2.2 Study population

Participants were consisted of frontline HCWs which was defined by clinical staff (e.g., doctors, nurses, and medical assistants) working at the medical, emergency, and anaesthesiology departments during the pandemic. In this study, The HCWs who worked at the medical and anaesthesiology departments were considered frontliners as a few medical wards have been dedicated as to isolate patients with COVID-19 during the pandemic, while one intensive care unit (under the anaesthesiology department) was assigned to provide supportive care for Category 5 COVID-19 patients who were critically ill. Additionally, HCWs must had at least one month of direct occupational exposure to patients diagnosed with COVID-19 infection during the pandemic. However, HCWs who were clinically diagnosed and actively receiving treatment and follow-up for any form of mental health disorder during the recruitment period were excluded. Also excluded were the HCWs who experienced non-occupational stressful event (e.g., death of first-degree relative due to COVID-19) one month prior to the recruitment. These exclusion criteria were listed in the beginning if the questionnaire. HCWs who did not qualify for the study were asked to discontinue the administration of the questionnaire and were thanked for their time.

### 2.3 Sample size estimation

Sample size was calculated using Epi Info (version 7.2.5) using the prevalence of depression, anxiety and stress (i.e., 24.3%, 25.8%, and 45.0%, respectively). We selected the prevalence of stress (i.e., 45.0%) for the sample size estimation as it is the largest number among the three disorders. With the prevalence of stress of 45.0%, confidence level of 95%, margin of error of 5%, and the finite population assumed to have 180 staff (i.e., 60 clinical staff from each department), the corrected sample size was 122 participants. When the sample size was inflated by 10% to account for non-response, the required final sample size was 135.

### 2.4 Sampling technique

A simple random sampling method was used to recruit the participants. Firstly, the researchers obtained the name list (i.e., sampling frame) of all HCWs working at the medical, emergency, and anaesthesiology departments. Each HCW (i.e., sampling unit) in the sampling frame was assigned a specific number. Using an online random number generator, 174 random numbers were generated. HCWs with the respective numbers were contacted to participate to the study. Once they agreed to participate, Google forms consisted of study objectives, instructions, questionnaires, and informed consents were disseminated to them via emails and/or WhatsApp contacts. HCWs who did not fulfil the inclusion criteria were replaced with other HCWs. The study was initiated in September 2021 and responses were collected until the fulfilment of the required sample size in September 2022.

### 2.5 Study instruments

Four self-administered questionnaires (in both English and Malay languages) combined in a single Google form were distributed to all selected participants. These include the sociodemographic questionnaire, Depression, Anxiety, and Stress Scale 21 (DASS-21), sources of workplace worries questionnaire, and Brief COPE inventory. A sociodemographic questionnaire was used to collect the sociodemographic data. This included questions about the participants’ age, gender, ethnicity, occupation, marital status, number of children, and living arrangement.

Depression, Anxiety, and Stress Scale (DASS-21) [16] or DASS-21 in short, is a self-administered questionnaire widely used to measure the severity of symptoms related to depression, anxiety and stress. It consists of 21 items that measures three domains (i.e., depression, anxiety, and stress). Participants were required to answer the DASS-21 using a 4-point Likert response, ranging from 0 (“never”) to 3 (“almost always”). Participants were then classified into different categories according to the severity of their symptoms (Table 1). The Malay version of the DASS-21 has previously been validated in Malaysia and demonstrates good reliability and validity [17].

**Table 1:** Scoring and interpretation of DASS-21.

Domains	Depression	Anxiety	Stress
Normal	0–4	0–3	0–7
Mild	5–6	4–5	8–9
Moderate	7–10	6–7	10–12
Severe	11–13	8–9	13–16
Extremely Severe	14+	10+	17+

Since there is no specific questionnaire to capture the sources of worries at workplace specific to COVID-19, we adapted and modified the questionnaire previously developed by Parthasarathy et al. [18]. This questionnaire consists of six items, including the HCWs’ worries of getting infected, infecting others, getting quarantined, inadequate personal protective equipment (PPE), inadequate training, and workload. Participants were required to indicate whether they were affected by the different sources of workplace worries using a “yes” and “no” dichotomous response. The English version of this questionnaire was forward and backward translated by a bilingual language secondary school teacher. Its face validity was established by three HCWs who worked at the same hospital.

The Brief-COPE inventory is a self-administered questionnaire to assess coping strategies used by individuals in response to stressful situations [19]. It consists of 28 items that measures 14 domains of coping (i.e., active coping, use of information support, positive reframing, planning, emotional support, venting, humour, acceptance, religion, self-blame, self-distraction, denial, substance use, and behaviour disengagement). Participants were required to rate the frequency of each coping strategy using a 4-point Likert response, ranging from 1 (“I haven't been doing this at all”) to 4 (“I've been doing this a lot”). Higher score in each of these domains indicates higher possibility of these coping strategies attribute to depression, anxiety, and stress. The Malay version of the Brief-COPE inventory has previously been validated in Malaysia and it demonstrated acceptable reliability and validity [20].

Up to three reminders were sent to the participants if they did not respond to the questionnaires within two months. If participants still did not response after the third reminder, they were deemed non-response. To avoid missing data, the Google form was pre-set in such a way that answers to all questions is mandatory prior to submission. To avoid duplicate responses, the researchers activated the “limit to one response” setting so that each participant can only participate once.

### 2.6 Statistical analyses

The Statistical Package for Social Science (SPSS) software (v 22.0) was utilised in the data analyses. Descriptive statistics such as frequency was used to describe categorical variables. Chi-square tests were conducted to determine the association between: (i) sociodemographic characteristics and depression, anxiety, and stress; and (ii) sources of workplace worries and depression, anxiety, and stress. Multiple linear regression analysis was performed to investigate the association between coping strategies and depression, anxiety, and stress. The significance level was set at p < 0.05.

### 2.7 Ethical considerations

The present study was approved by the Ethics Committee of the National University of Malaysia (FF-2022-142). Information regarding the objectives of the study was provided to the potential participants and they were assured of the confidentiality of the collected data. To ensure privacy and anonymity of the participants, the Google forms was preset in such a way that email addresses of the participants were not collected. In addition, participants were also reminded that it is their right to refuse to participate to the present study if they feel uncomfortable. A disclaimer was made on the first page of the questionnaire, stated that participants who reported high level of depression, anxiety, and stress will not be identified and notified to the hospital management but were highly encouraged to seek for help whenever possible. Informed consent from the participants was obtained digitally via Google forms.

## 3 Results

A total of 137 HCWs (out of 174) responded to the questionnaire, resulted in a 78.7% response rate. Among the 137 participants, a majority were young adult aged 20–35 (73.0%), females (81.8%), Malays (81.0%), and nurses (51.1%). With regards to their personal lives, most of them were married (51.1%), having children (58.4%), and living with family or at home (73.7%). This information is tabulated in Table 2.

**Table 2:** Sociodemographic characteristics of the participants.

Sociodemographic characteristics	N (%)
Age	
20–35	100 (73.0)
>36	37 (27.0)
**Gender**	
Male	25 (18.2)
Female	112 (81.8)
**Race**	
Malay	111 (81)
Non-Malay	26 (19)
**Occupational job**	
Nurse	70 (51.1)
Non-nurse	67 (48.9)
**Marital status**	
Single	67 (48.9)
Married	70 (51.1)
**Having children**	
Yes	57 (41.6)
No	80 (58.4)
**Living arrangement**	
Living alone or at hostel	36 (26.3)
Living with family or at home	101 (73.7)

The prevalence of depression, anxiety, and stress was 69.3%, 77.4%, and 57.7%, respectively (Table 3). Among the participants who suffered from depression and stress, a majority of them were having moderate symptoms. A large proportion of the participants who were having anxiety, however, experienced extremely severe symptoms.

**Table 3:** Prevalence of depression, anxiety, and stress among the participants.

	N, (Prevalence)				
	Mild	Moderate	Severe	Extremely Severe	Total
**Depression**	13 (9.49)	40 (29.20)	16 (11.68)	26 (18.98)	95 (69.34)
**Anxiety**	15 (10.95)	21 (15.33)	17 (12.41)	53 (38.69)	106 (77.37)
**Stress**	16 (11.68)	26 (18.98)	23 (16.79)	14 (10.22)	79 (57.66)

Chi-square tests were conducted to determine association between sociodemographic characteristics and depression, anxiety, and stress (Table 4). Of note, none of the sociodemographic characteristics is associated with depression, anxiety and stress.

**Table 4:** Association between sociodemographic characteristics and depression, anxiety, and stress.

Sociodemographic Characteristics	Depression		Anxiety		Stress	
	N	P-value	N	P-value	N	P-value
**Gender**		0.872		0.476		0.478
Male	17		18		16	
Female	78		88		63	
**Age**		0.784		0.773		0.603
20–35	70		78		59	
>36	25		28		20	
**Race**		0.338				0.662
Malay	79		87	0.561	65	
Non-Malay	16		19		14	
**Marital Status**		0.841		0.732		0.900
Single	48		51		39	
Non-Single	47		55		40	
**Having Children**		0.342		0.966		0.512
Yes	37		44		31	
No	58		62		48	
**Living Arrangement**		0.663		0.946		0.766
Living alone or at hostel	26		28		20	
Living with family or at home	69		78		59	
**Occupational Job**		0.588		0.246		0.826
Nurse	50		57		41	
Non-nurse	45		49		38	

Chi-square tests were also performed to determine the association between the sources of workplace worries and depression, anxiety, and stress symptoms (Table 5). It was shown that depression was associated to all sources of workplace worries, except “fear of getting infected” (p = 0.089). Meanwhile, our data demonstrate that anxiety and stress were associated to all sources of workplace worries.

**Table 5:** The association between sources of workplace worries and depression, anxiety and stress.

Sources of Workplace Worries	Depression		Anxiety		Stress	
	N	P-value	N	P-value	N	P-value
**Fear of getting infected**		0.089		0.011*		0.002*
Yes	60		68		55	
No	35		38		24	
**Fear of infecting others**		0.020*		0.001*		0.001*
Yes	71		80		63	
No	24		26		16	
**Inadequate PPE**		0.008*		<0.001*		<0.001*
Yes	55		62		51	
No	40		44		28	
**Getting quarantined**		0.005*		0.026*		0.001*
Yes	61		65		54	
No	34		41		25	
**Inadequate training**		0.001*		0.011*		<0.001*
Yes	62		65		54	
No	33		41		25	
**Workload**		0.001*		0.001*		<0.001*
Yes	81		89		70	
No	14		17		9	

Multivariable linear regression was conducted to identify the correlation between the various coping strategies and the levels of depression, anxiety, and stress (Table 6). It was found that humour (β = 0.821), self-blame (β = 0.686), denial (β = 0.676), substance use (β = 0.835), and behavioural disengagement (β = 0.583) were positively correlated to depression. However, active coping (β = −0.648) and acceptance (β = −0.602) were negatively correlated to depression. On the other hand, active coping (β = 0.913), planning (β = 0.879), acceptance (β = 0.831), religion (β = 0.704), and self-distraction (β = 0.929) were positively correlated to stress, with only substance use (β = −0.417) negatively correlated to stress. All coping strategies did not correlate with anxiety.

**Table 6:** Multivariable linear regression to identify significant coping strategies in depression, anxiety, and stress.

Coping Strategies	Depression		Anxiety		Stress	
	β	P-value	β	P-Value	β	P-Value
Active Coping	−0.648	0.010*	0.158	0.418	0.913	<0.001*
Use of Informational Support	−0.072	0.775	0.361	0.069	0.190	0.401
Positive Reframing	−0.144	0.589	0.152	0.466	0.359	0.133
Planning	−0.446	0.071	0.027	0.888	0.879	<0.001*
Emotional Support	0.160	0.533	0.309	0.126	−0.021	0.927
Venting	0.498	0.031	−0.003	0.988	0.110	0.589
Humour	0.821	0.001*	0.102	0.577	−0.381	0.070
Acceptance	−0.602	0.029*	−0.108	0.615	0.831	0.001*
Religion	−0.415	0.129	−0.064	0.765	0.704	0.004*
Self-Blame	0.686	0.001*	−0.027	0.866	0.034	0.853
Self-Distraction	−0.345	0.175	−0.206	0.300	0.929	<0.001*
Denial	0.676	0.004*	0.220	0.222	−0.313	0.128
Substance Use	0.835	<0.001*	0.189	0.274	−0.417	0.036*
Behavioural Disengagement	0.583	0.004*	−0.015	0.925	0.149	0.407

β = coefficient; SE = standard error of coefficient; * indicates significant p-value (<0.05).

## 4 Discussion

According to our findings, we found that the prevalence of depression, anxiety, and stress among frontline HCWs during the pandemic was 69.3%, 77.4%, and 57.7%, respectively. These findings were higher compared to previous studies, in which a recent systematic review reported 24.3%, 25.8%, 45.0% of the HCWs suffered from depression, anxiety, and stress, respectively [5]. On top of that, the depression, anxiety, and stress in our study were also more prevalent [12] and more severe [21] when compared to other local studies. Such inconsistencies could be attributed to the differences in study populations and study instruments. Given that all our participants were strictly frontline HCWs who served in the medical, emergencies, and anaesthesiology departments and provided direct care to COVID-19 positive patients on a daily basis, it is not surprising that our HCWs were more prone to depression, anxiety, and stress. In addition, with the use of DASS-21 in the present study, we took all levels of depression, anxiety, and stress into account when determining their prevalence, including those under the “mild” and “moderate” categories. Not surprisingly, our prevalence of depression, anxiety, and stress is higher than other studies.

According to the findings from previous research, female HCWs were associated with higher levels of depression, anxiety, and stress due to higher role pressure, challenges in maintaining work-life balance, and lack of adequate support [14,15]. Some researchers also reported that being married and having children were significant predictors of depression, anxiety, and stress during the pandemic [[22], [23], [24]]. Although a majority of our participants were female, married, and living at home, such trends were not observed in the present study. This could be due to the fact that the Malaysian government implemented a series of “lock down period” (also known as “movement control order”) during the pandemic. Hence, the spouse of these female frontline HCWs can stay at home and take up the responsibility to look after the family. Additionally, the Ministry of Health, Malaysia also provided the COVID-19 financial aid to all frontline HCWs who risked their lives during the pandemic, easing their economic burden at this trial time [25]. Such relieving factors explain the low psychological impact from sociodemographic profile in the present study.

In fact, our participants were more affected by the various sources of workplace worries during the pandemic, such as the fear of getting infected, fear of infecting others, inadequate PPE, getting quarantined, inadequate training, and workload. The fear to COVID-19 infection and passing the infection to others, especially at the time when COVID-19 vaccination was unavailable, were common findings reported by previous studies [26]. Shortages of PPE in the early phases of the pandemic was a real struggle in many developing countries, including Malaysia [27]. Given that PPE is the last “line of defense” in the hierarchy of control, many of our HCWs found themselves helpless and vulnerable when they were forced to use the same sets of PPE for a prolonged period of time, eventually leading to depression, anxiety, and stress. This finding was in agreement to the previous research [28]. In addition, the lack of social connection, long-term isolation from family members and beloved people, and the lack of support during the quarantine period can lead to feelings of loneliness, which in turn resulted in depression, anxiety, and stress [29]. Furthermore, frontline HCWs always work under intense pressure for prolonged hours due to the high workload. Worst still, unlike the Ministry of Health hospitals which are able to mobilise staff between facilities according to human resource need, our hospital, which is under the governance of the Ministry of Higher Education, did not have the privilege of such staff mobilisation from the other health care facilities. As a result, our participants have to deal with extra workload and extra work shifts during the surge of COVID-19 cases and this is a worry that eventually transformed to depression, anxiety, and stress.

In terms of coping strategies, it was found that active coping and acceptance were preventive factors of depression. Active coping, defined as proactive measures taken to eliminate or mitigate the stressors, has been proven to effectively reduce depression as it helps the individual to focus on future, expand personal resources, establish goals, and putting efforts to achieve these goals [30]. Conversely, other coping strategies such as self-blame, denial, substance use, and behavioural disengagement were predictors of depression. This finding is agreed by a previous study which reported that self-blame, denial, substance use, and behavioural disengagement increased the odds of depression by 1.57, 2.57, 3.33, 2.31, respectively [31]. To our surprise, humour was associated with a higher risk of depression. This could be explained by the fact that some HCWs attempted to use humour (including sarcasm) to “mask” their grief and frustration when experiencing depressing events [31].

One finding that caught our attention is that substance use has shown to be a protective factor towards stress. Although substance use (including alcohol) can temporarily avoid life stress and negative emotions, it is only beneficial in short-term. Considering the COVID-19 pandemic is a chronic stressor, this strategy would lead to more distress in long term [32]. While planning can be a helpful coping strategy in many situations, it can contribute to stress when it is not effectively managed or when it is overly rigid. Overly rigid planning can be problematic when unexpected events or changes occur, which is a common situation during the pandemic. If HCWs are unable to adapt to new information or situations, they may experience stress when their carefully laid-out plans are disrupted. Generally, acceptance is associated with reducing stress. However, due to the time constrain, frontline HCWs often do not have time to seek help, guidance, or counselling and they simply accept their stressful situation. As a result, they may miss out on valuable resources for managing stress. Furthermore, instead of facing and addressing the source of stress, frontline HCWs who rely on self-distraction tend to avoid dealing with the issues directly. This avoidance can prevent them from finding effective solutions or making necessary changes, perpetuating the stressors.

Although the COVID-19 has shifted to an endemic phase [33], the findings of the present study can still improve healthcare services in two ways. Firstly, the findings of this study help hospital management to understand the importance of providing evidence-based education and sufficient PPE to relieve workplace worries among the HCWs, in preparation for the next surges of COVID-19 cases or future pandemic. Secondly, given that some HCWs utilised negative coping strategies during the pandemic, our study highlights that proper mental health services, including support groups and psychosocial therapies, ought to be prepared for this group of vulnerable workers so that they adapt the correct coping skills to prepare for the next pandemic.

## 5 Strengths and limitation

One of the major strengths of the present study is that this was the first study that investigated the impact of COVID-19 on frontline HCWs in Malaysia. As such, it sheds light on the considerable levels of depression, anxiety, and stress experienced by this specific group, contrasting them with the broader population of HCWs during the pandemic. Secondly, the participants were not required to administer the questionnaire physically (i.e., face-to-face) with the researchers. This allowed them to provide the most honest responses possible on sensitive issues and, hence, avoid social desirability bias.

Nonetheless, this study is limited by the relatively small number of sample size. Secondly, since our data collection was conducted over a long period of time (i.e., a one-year duration), the varying trends of the pandemic over the year may have changed the psychological perceptions and impacts among the frontline HCWs. Thirdly, the use of self-reported screening for inclusion and exclusion criteria (via the online questionnaire) may lead to underestimation or overestimation of depression, anxiety, and stress as compared to screening done by the researchers. Furthermore, it was previously found that contact-based HCWs were more mentally impacted due to job security and uncertainty of career pathway, unstable future income, and high competition at workplace [34]. However, the association between contract job and the levels of depression, anxiety, and stress were not captured in our study.

## 6 Recommendation

In order to enhance the findings of the study, several recommendations for future research should be considered. Firstly, expanding the sample size could significantly bolster the statistical power and generalisability of the results. A larger and more diverse sample can capture a broader spectrum of perspectives and potentially reveal subtler relationships. Additionally, it may be beneficial to contemplate the necessity of a nationwide study to ensure the findings hold true across different regions or demographics in Malaysia, thereby increasing the external validity of the research.

From a clinical practice point of view, given the high prevalence of depression, anxiety, and stress among the frontline HCWs in Malaysia, a more thorough risk assessment ought to be conducted to identify all potential hazards and exposure at the workplace. Proper standard operating protocols (SOP) for quarantine must be stated clearly in the hospital policy. In addition, HCWs shall be offered sufficient emotional support during the quarantine period. Appropriate on-the-job training must be provided to all HCWs, especially those working at the medical, emergency, and anaesthesiology departments to ensure occupational safety and health. The hospital management may increase the human resource and allow departmental rotation to avoid excessive workload among the frontline HCWs. Last but not least, adequate PPE of acceptable quality shall be ensured, with the priority given to the vulnerable departments.

## 7 Conclusion

To our knowledge, this is the first study evaluating the occupational and psychological impacts of the COVID-19 pandemic among the frontline HCWs in Malaysia. We found that the prevalence of depression, anxiety and stress is high in this population, which could be attributed by the various sources of workplace worries and the inappropriate coping strategies among the frontline HCWs. Hence, effective measures need to be implemented promptly to minimise the workplace worries and inappropriate coping strategies among the frontline HCWs before the health care system is hit by another wave of COVID-19 pandemic.

## Author contribution statement

Halim Ismail: Conceived and designed the experiments; Contributed reagents, materials, analysis tools or data. Yong Hong Phan; Kausalyaa Chandriah; Mohd Ridzwan Arman: Performed the experiments. Nurul Nabihah Mokhtar; Siti Aishah Hamdan: Analyzed and interpreted the data. Sheng Qian Yew: Analyzed and interpreted the data; Wrote the paper.

## Data availability statement

Data will be made available on request.

## Declaration of competing interest

The authors declare that they have no known competing financial interests or personal relationships that could have appeared to influence the work reported in this paper.
